# Pathogenic Potential of Two Sibling Species, *Anisakis simplex* (s.s.) and *Anisakis pegreffii* (Nematoda: Anisakidae): *In Vitro* and *In Vivo* Studies

**DOI:** 10.1155/2015/983656

**Published:** 2015-01-21

**Authors:** Chan-Hyeok Jeon, Jeong-Ho Kim

**Affiliations:** Department of Marine Bioscience, Gangneung-Wonju National University, Gangneung 210-702, Republic of Korea

## Abstract

The pathogenic potentials of two sibling nematodes* Anisakis simplex *sensu stricto (s.s.) and* A. pegreffii* were compared by* in vitro* and* in vivo* studies. Live third-stage larvae of each species were subjected to agar blocks made using PBS or RPMI-1640, overlaid with different supernatants (artificial gastric juice, PBS, and RPMI-1640), and their penetration ability was compared. Their tolerance of artificial gastric juice was also tested. Further, they were introduced into rats by gastric intubation, and the* in vivo* locations of them were investigated.* A. pegreffii* showed higher penetration ability than* A. simplex* (s.s.) in most of the experimental conditions, except for the RPMI-1640 agar block overlaid with artificial gastric juice. In an acid tolerance test, the mean survival times were 6.1 days for* A. simplex* (s.s.) and 4.2 days for* A. pegreffii*. In an animal experiment,* A. simplex* (s.s.) stayed for a shorter time in the stomachs of rats than* A. pegreffii*. Some* A. pegreffii* and* A. simplex* (s.s.) were embedded in the gastric mucosa or freely existed in the abdominal cavity. All of these results suggest that* A. pegreffii* has the pathogenic potential to cause anisakidosis in humans when ingested, as does* A. simplex* (s.s.).

## 1. Introduction

Anisakidosis is one of the most important fish-borne zoonoses worldwide [1]. It occurs from the human ingestion of raw or undercooked fish or cephalopods harboring the third-stage larvae (L3) of nematodes belonging to family Anisakidae. Although a number of species belonging to this family are known to cause human infection, genus* Anisakis* and genus* Pseudoterranova* are the most frequently involved, and species of the genus* Anisakis* are considered to be the most common cause of human infections [2].

Anisakidosis is obviously associated with the custom of eating raw or undercooked marine fish or cephalopods, which explains why over 90% cases of anisakidosis are reported in Japan, with most of the rest from European countries [1, 2]. However, anisakidosis occurs worldwide and the number of cases from other countries has been increasing recently, as diagnostic methods improve and culinary habits change [2, 3]. Live L3 anisakid nematodes can cause human infection called anisakidosis or anisakiasis, and their symptoms include mild to severe abdominal pains, nausea, vomiting, and diarrhea. In addition, allergic reactions are found in some cases [2].

Anisakid nematodes are known to have low host specificity in the larval stage, and, in fact, more than 200 fish and 250 cephalopods species have been reported to harbor anisakid nematode larvae [3]. However, each* Anisakis* species has a specific distribution area and host range, depending on the availabilities of its intermediate and final hosts. For example,* Anisakis simplex* sensu stricto (s.s.) is widespread between 35°N and the Arctic Circle.* A. pegreffii *is distributed in the Mediterranean Sea and the Austral region between 30°N and 55°S.* A. simplex *C has a discontinuous range, including the Canadian and Chilean Pacific coasts, New Zealand waters, and the South African Atlantic coast [4]. From an epidemiological point of view, it is necessary to clearly understand the distribution of each* Anisakis* species and its teleost hosts because not all* Anisakis* species are dangerous to humans, as mentioned by Klimpel and Palm [5].

The particular source of infection varies depending on the fish eating customs of the country. In Japan, the main fish species responsible for anisakidosis are chub mackerel (*Scomber japonicus*) and common squid (*Todarodes pacificus*). In Western Europe, herring (*Clupea harengus*) and anchovy (*Engraulis encrasicolus*) are the main species involved [2]. These are due to the availabilities of infected teleost hosts, which can be consumed as raw or undercooked fish by humans in each area.

To diagnose anisakidosis, determining the history of what fish or cephalopod species were eaten, along with the occurrence of typical symptoms such as abdominal pain, vomiting, and nausea, provides important clues. Surgical or gastroscopic removal and identification of the larvae provide a definitive diagnosis [6]. The most commonly involved anisakid nematode species are known to be* A. simplex* s.l. and* Pseudoterranova decipiens* s.l. Other species, including* A. physeteris *and* Contracaecum* sp., are known to be less commonly responsible for human infection [2, 3, 5]. However, most nematodes isolated from human cases have been conventionally diagnosed as* A. simplex* or* Anisakis* sp., without further identification at the species level, so some of them might have been misdiagnosed. In Japan, most human infections are known to be due to* A. simplex* (s.s.), although* A. pegreffii* is known to be frequent in the East China Sea and the Sea of Japan (The East Sea in Korea) [7–9]. However,* A. pegreffii* is also thought to be able to cause problems in humans when ingested, and, in fact, several cases of anisakidosis due to* A. pegreffii* have been reported in Italy [10–12]. Arizono et al. [13] mentioned that the dominance of* A. simplex* (s.s.) over* A. pegreffii* as the causative agent of anisakidosis in Japan may be due to pathogenic differences between these two* Anisakis* species and demonstrated that* A. simplex* (s.s.) has the potential to survive acidic gastric juice to some extent and penetrate human gastrointestinal mucosa. However, it is still unclear what mechanisms work behind these two* Anisakis* species in terms of pathogenicity against humans.

In this study, we compared the penetration ability of* A. simplex* (s.s.) and* A. pegreffii *L3 in various* in vitro* conditions simulating the physiological conditions of human stomachs. In addition, their tolerance against artificial gastric juice was tested and compared. We also intubated live* A. simplex* (s.s.) and* A. pegreffii* L3 into Sprague-Dawley male rats to compare their invasion ability* in vivo*.

## 2. Materials and Methods

### 2.1. Anisakid Nematode Collection

*Anisakis simplex* (s.s.) L3 used in this study were collected from chum salmon (*Oncorhynchus keta*, mean body length: 60.3 cm and mean body weight: 1,090 g) that migrated to the Namdae River in eastern Korea for spawning in December 2012.* A. pegreffii* L3 were collected from chub mackerel (*Scomber japonicus*, mean body length: 36.3 cm and mean body weight: 699.8 g) bought from a local fish market in Busan, southern Korea in December 2012. Our previous studies demonstrated that chum salmon and chub mackerel are the main hosts of* A. simplex* (s.s.) and* A. pegreffii*, respectively, in Korea [14, 15]. All the collected nematodes were examined by a stereomicroscope and actively moving nematode larvae without any injury were maintained in sterile PBS at room temperature until use. All the experiments were conducted within 1 h after isolation.

### 2.2. *In Vitro* Penetration Ability Test

Agar block plates were made according to the methods of Arizono et al. [13], with some modifications. Two different agar solutions were made for the experiment: 0.75% agar (Bacto, USA) in PBS (pH 7.2) (Group A) and 0.75% agar in RPMI 1640 medium solution (pH 4.0, Sigma, USA) with 20% FBS (Lonza, USA) (Group B). Two mL of one of the solutions was poured into each well of a 12-well plate (SPL, Korea), to make the agar block plate. Then, 100 *μ*L of one of 3 different supernatants was placed onto each well: PBS (pH 7.2), artificial gastric juice (AGJ; 0.1% commercial pepsin, 0.1% porcine stomach mucin (Sigma, USA), 0.12% NaCl, 0.02% KCl; pH 1.8 adjusted with HCl), and RPMI-1640 (RPMI-1640, 20% FBS, 1% commercial pepsin, pH 4.0). Twelve actively moving individuals of either* A. simplex* (s.s.) or* A. pegreffii* L3 were placed on each well, and the well plates were incubated at 37°C under a 5% CO_2_ atmosphere for 48 h. The experimental design is summarized in Table 1. The number of L3 that penetrated into the solid agar block was counted periodically during incubation (at 1, 12, 24, and 48 h) and all the experimental groups were replicated five times. All the supernatants were replaced with new ones every 12 h to prevent evaporation and pH changes. If more than half of the nematode's body penetrated into the agar block, it was considered to have penetration ability. All the nematodes were recovered after the experiment and subjected to molecular identification using PCR-RFLP with subsequent sequencing.

### 2.3. Acid Tolerance Test

Artificial gastric juice (0.1% pepsin, 0.1% porcine stomach mucin, 0.12% NaCl, 0.02% KCl; pH 1.8) was used to compare the survival of 2 different* Anisakis* species L3 in acidic conditions that mimic the human stomach, and PBS (pH 7.2) was used for comparison. Ten actively moving* A. simplex *(s.s.) or* A. pegreffii* L3 were incubated in 20 mL of artificial gastric juice or PBS in a Petri dish at 37°C in a 5% CO_2_ atmosphere for 7 days, and the medium was replaced every 24 h. All the experimental groups were triplicated. Dead larvae were removed during the experiments and stored in 70% alcohol for further molecular identification. After 7 days, all the surviving nematodes were subjected to molecular identification at the species level.

### 2.4. Animal Ethics

All the experiments were carried out in accordance with the regulations for animal experiments at Gangneung-Wonju National University (approval number: GWNU-2012-23).

### 2.5. *In Vivo* Experimental Infection

Sprague-Dawley male rats (7 weeks old, purchased from Samtako, Korea) were used for experimental infection with live L3. Twenty-four male rats (mean body weight: 280 ± 10 g) were kept for one week in an animal housing facility of Gangneung-Wonju National University and starved for 12 h before starting the experiment. Anesthesia was induced by injection of tiletamine-zolazepam (40 mg/kg, Zoletil 50, Virbac, Korea) and xylazine hydrochloride (5 mg/kg, Rompun, Bayer, Korea) to thigh muscles of each individual. Actively moving* A. simplex* (s.s.) or* A. pegreffii* L3 in 5 mL of sterile PBS were then introduced into the gastric lumen of each rat using a gastric tube, as described by Figueiredo Jr. et al. [16]. Twelve rats were used for* A. simplex* (s.s.) and* A. pegreffii*, respectively, and 10 L3 of either* A. simplex* (s.s.) or* A. pegreffii* were introduced into an individual rat. Three rats were euthanized by excessive CO_2_ gas at 3, 6, 12, and 24 h after administration. The locations of* Anisakis* larvae in each rat were recorded during necropsy, and all of the recovered larvae were washed with sterile PBS and placed individually into Eppendorf tubes at −20°C for further molecular analysis.

### 2.6. Molecular Identification of L3 Anisakid Nematodes

PCR-RFLP was conducted to identify all of the* Anisakis* L3 used in this study at the species level. DNA was extracted by the method described by Wasko et al. [17]. PCR amplification of rDNA fragment ITS1-5.8S-ITS2 was conducted with the primer set A-B [18]. Amplification was conducted using MyCycler (Biorad, USA), with the following conditions: denaturation at 94°C for 10 min and then 35 cycles at 94°C for 40 s, 54°C for 40 s, and 72°C for 90 s, and postamplification at 72°C for 7 min. The digestion of PCR products with restriction enzymes* Hha*I and* Hinf*I was conducted by the method described by D'Amelio et al. [18]. The digested products were analyzed by electrophoresis in 1.5% agarose gel containing ethidium bromide (Sigma, USA). Pictures were taken using the Gel Logic 100 Imaging System (Biostep, Germany) supported by Kodak Molecular Imaging Software version 4.0. The amplified products of the ITS region were purified using the AccuPrep Gel Purification Kit (Bioneer, Korea) according to the manufacturer's instructions. The purified PCR products were directly sequenced using the ABI Prism 3730 XL DNA Analyzer (PE Applied Biosystems, USA). The obtained sequences were aligned and compared with the published sequences in the GenBank database (NCBI) using Clustal W [19].

### 2.7. Statistical Analysis

All data are expressed as mean ± standard errors. Statistical analyses were carried out using SPSS 20.0 software (SPSS Inc., Chicago, Illinois, USA). A value of *P* < 0.05 was considered statistically significant in all the experiments except for acid tolerance test (*P* < 0.01). Data of* in vitro *penetration ability test and* in vivo* experimental infection were analyzed by one way ANOVA followed by Duncan's multiple range tests or independent *t*-test. Survival rates of two* Anisakis* species in acid tolerance test were determined using the Kaplan-Meier survival method and differences between groups were identified using log-rank analysis.

## 3. Results

### 3.1. *In Vitro* Penetration Ability Test of* A. Simplex* (s.s.) and* A. pegreffii*

For* A. simplex* (s.s.), there were no significant differences among all the experimental groups until 24 h after administration. As time progressed, the penetration rate increased among all the experimental groups and, in particular, significantly higher penetration rates were observed in Group B (agar block in RPMI-1640) with AGJ supernatant at 48 h than in Group A (agar block in PBS) with the same supernatant (*P* < 0.05, Duncan's multiple range tests) (Table 1).

For* A. pegreffii*, a significantly higher penetration rate was found in Group A (agar block in PBS) with AGJ compared to Group B (agar block in RPMI-1640) with the same supernatant (*P* < 0.05, Duncan's multiple range tests) (Table 1). As time progressed, the penetration rates increased in all the experimental groups but most of them were not significant.

When the penetration rates of* A. simplex* (s.s.) and* A. pegreffii* in each experimental groups were compared, there were no significant differences between them in Group A with PBS supernatant. However, the penetration rates of* A. pegreffii* were significantly higher than those of* A. simplex *(s.s.) in all the other experimental groups at 1~24 h, depending on the experimental conditions (*P* < 0.05, independent *t*-test) (Table 2). In particular, significant different penetration rates between* A. simplex* (s.s.) and* A. pegreffii* were consistently observed in Group A with AGJ supernatant. In case of Group B with AGJ supernatant, however,* A. simplex* (s.s.) showed significantly higher penetration rate than* A. pegreffii* in the same condition at 48 h (*P* < 0.05, independent *t*-test) (Table 2).

### 3.2. Acid Tolerance Test

**Figure 1:**
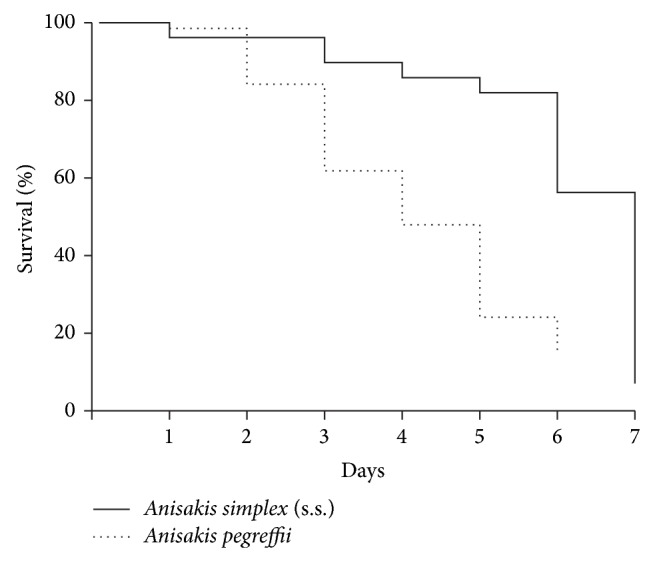
Kaplan-Meier survival curves showing survival rates of* Anisakis simplex* (s.s.) (solid line) and* Anisakis pegreffii* (dotted line) in AGJ. Data are mean values. Statistical significance was determined by log-rank test. The percentage of survival for* A. simplex* (s.s.) was significantly greater than that for* A. pegreffii* (*P* = 0.001). AGJ: artificial gastric juice.

The survival of* Anisakis* larvae was measured in artificial gastric juice (pH 1.8) at 37°C under a 5% CO_2_ atmosphere. In the acidic environment,* A. simplex* (s.s.) started to die at 1 DPI (days after immersion) and the survival rate gradually decreased every day during the experiment. In particular, it drastically decreased during 6-7 DPI (Figure 1, Table 3).* A. pegreffii *showed a 100% survival rate on 1 DPI but started to die at 2 DPI, and the survival rate drastically decreased to 0% at 7 DPI (Figure 1, Table 3). As shown in Figure 1, the Kaplan-Meier survival curves demonstrated that the survival rate of* A. simplex* (s.s.) was significantly greater than that of* A. pegreffii* (*P* = 0.001 by the log rank test). The mean survival times were 6.1 days for* A. simplex* (s.s.) and 4.2 days for* A. pegreffii*. In PBS conditions, the mean survival rate was 80.0 ± 15.3% for* A. simplex* (s.s.) at 7 DPI but 96.7 ± 3.3% for* A. pegreffii *at 7 DPI (Table 3).

### 3.3. *In Vivo* Experimental Infection

After 30* A. simplex* (s.s.) L3 were administered to rats, 86.7% (26/30) were recovered from 3 rats at 3 h after administration (Table 4). 60.0% (18/30) were found freely in the stomach and significantly higher than in other body parts of rats (*P* < 0.05, Duncan's multiple range test). As time progressed, the percentage of L3 found freely in the stomach significantly decreased (*P* < 0.05, Duncan's multiple range test) and the percentage of the recovered L3 after necropsy between 3 h and 24 h was significantly different (*P* < 0.05). At 24 h, 20.0% (6/30) of the administered L3 were recovered from 3 rats, and no L3 were found freely in the stomach (Table 4). For the number of* A. simplex* (s.s.) L3 in other organs, there was no statistical difference at different time points. When 30* A. pegreffii* L3 were administered, 73.3% (22/30) were recovered at 3 h after administration (Table 4). 50% (15/30) were found freely in the stomach and significantly higher than in other body parts of rats (*P* < 0.05, Duncan's multiple range test). As time progressed, the percentage of the recovered L3 decreased, but was not statistically significant. For the number of* A. pegreffii* L3 in other organs, there were no statistical differences at each time point (Table 4). No statistical differences were found in the percentage of organ distributions between two* Anisakis* species at each time point (*P* > 0.05, independent *t*-test, data not shown). In addition, there were no statistical differences in the number of recovered nematodes between two different* Anisakis *species at four different time points during experiment (*P* > 0.05, independent *t*-test, data not shown).

### 3.4. PCR-RFLP Analysis

**Figure 2:**
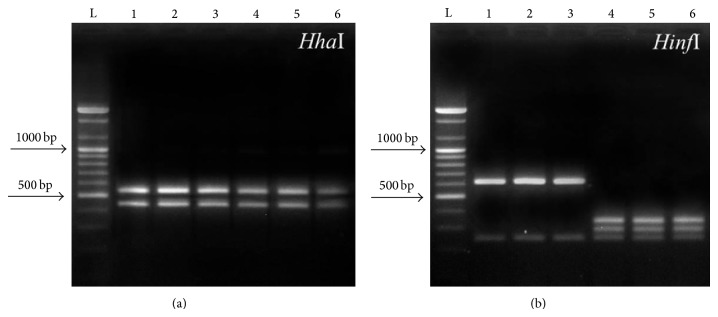
PCR-RFLP profiles of third-stage anisakid nematode larvae used in this study. PCR-amplified ITS region with* Hha*I (a) and* Hinf*I (b) restriction enzymes. (L: Ladder, 1–3:* Anisakis simplex* (s.s.) isolated from chum salmon, and 4–6:* Anisakis pegreffii* isolated from chub mackerel.)

PCR-amplified products were treated with two different restriction enzymes. When* Hha*I was applied to the PCR product of* Anisakis* L3, all of the individual samples showed the same banding pattern: 550–430 bps. When they were treated with* Hinf*I, a 620–250 bps banding pattern was observed in all of the* Anisakis* larvae isolated from chum salmon, and a 380–300–250 bps banding pattern was observed in all of the* Anisakis* larvae isolated from chub mackerel (Figure 2). These patterns corresponded to the previously known patterns of* A. simplex* (s.s.) and* A. pegreffii*, respectively. In addition, all of the sequenced PCR-amplified products of nematode samples from chum salmon and chub mackerel corresponded to those of* A. simplex* (s.s.) and* A. pegreffii *registered in the NCBI, respectively (data not shown).

## 4. Discussion

Anisakidosis is one of the most important zoonoses worldwide, especially in countries where eating raw fish is a custom. More than 2,000 cases of anisakidosis are reported every year worldwide, and more than 90% of these are from Japan, which is thought to be due to their custom of eating a variety of raw or undercooked fish as sashimi or sushi [1, 2, 10, 13]. However, the occurrence of anisakidosis in other countries has been increasing due to the rising popularity of eating raw or undercooked fish as well as improved diagnostic techniques [2, 3]. Korean people have also traditionally eaten raw fish and anisakidosis has been frequently reported since 1971 [20, 21].

*Anisakis simplex *s.l. is the most commonly implicated species in anisakidosis, while* Pseudoterranova decipiens* s.l. is less frequent, but still common [2]. Other species seem to be rarely involved, and some cases need to be investigated further. Recently, several cases in Italy have indicated that* A. pegreffii *can also be a major concern in countries where it is dominant [10–12], as* A. simplex* (s.s.) is in Japan [7, 13]. Although* A. simplex* (s.s.) and* A. pegreffii* sympatrically occur in several countries, including Japan,* A. simplex* (s.s.) is known to be the more dominant cause of anisakidosis in Japan [6] and anisakidosis due to* A. pegreffii* has been rarely reported in other countries so far. Suzuki et al. [8] and Arizono et al. [13], using the agar block method, suggested that* A. simplex* (s.s.) has more potential to survive in acidic conditions and is more invasive than* A. pegreffii in vitro*.

In this study, we designed two different agar blocks with three different supernatants: an agar block made with PBS (Group A) or RPMI-1640 (Group B) overlaid with AGJ, PBS, or RPMI-1640. RPMI-1640 has been used for culturing many nematode species, including* Anisakis *species [22]. Thus, we used RPMI-1640 with 20% FBS to make agar blocks that mimic the physiological conditions of human gastrointestinal tracts. Moreover, AGJ was overlaid to make conditions similarly acidic to the human stomach, and two other solutions were used for comparison.

When two* Anisakis* species were subjected to these 6 different experimental conditions, they showed different penetration abilities. The penetration ability of* A. pegreffii* was significantly higher than that of* A. simplex* (s.s.) in most of the experimental conditions except for Group A with PBS supernatant (*P* < 0.05, independent *t*-test) (Table 2). However, in Group B overlaid with AGJ, which is thought to have the most similar environmental conditions to the human stomach in this study,* A. simplex *(s.s.) showed significantly higher penetration ability than* A. pegreffii* after 48 h (*P* < 0.05, independent *t*-test) (Table 2).

Similar studies have been conducted by Suzuki et al. [8] and Arizono et al. [13]. Their studies demonstrated that* A. simplex* (s.s.) penetrated the agar block at a significantly higher rate than* A. pegreffii*, while* A. pegreffii* showed similar or higher penetration ability than* A. simplex* (s.s.) in most experimental groups in this study. However, the experimental conditions were different; agar blocks made with a physiological saline solution were used in their studies, while agar blocks made with PBS or RPMI-1640 were used in this study to make conditions similar to human gastrointestinal tracts. In addition, the L3 nematodes were immersed with AGJ and then immediately placed on the agar block in their study [13], while 10 *μ*L of AGJ was overlaid on the agar block during the experiment to mimic the acidity of human stomachs in this study. In Group B (agar block in RPMI-1640) with AGJ, which is thought to be the most similar to the physiological conditions of the human stomach,* A. simplex* (s.s.) showed significantly higher penetration ability than* A. pegreffii* at 48 h (Tables 1 and 2). However, in Group A (agar block in PBS) with AGJ,* A. pegreffii *showed consistently higher penetration ability than in Group A with other supernatants during the experimental period (Tables 1 and 2). It seems that acidic conditions are a harsher environment for* A. pegreffii* than* A. simplex* (s.s.), and* A. pegreffii* is thought to more actively evade acidic conditions than* A. simplex* (s.s.) in Group A (agar block in PBS) with AGJ. In addition, Group B (agar block in RPMI-1640) with AGJ is thought to be more a tolerable environment for* A. pegreffii* than Group A (agar block in PBS) with AGJ, resulting in a lower penetration rate than* A. simplex* (s.s.). The penetration ability of* A. pegreffii* seems more susceptible to environmental changes than that of* A. simplex* (s.s.).

In the acid tolerance test,* A. simplex *(s.s.) showed a significantly higher survival rate and also had a longer mean survival time than* A. pegreffii *(6.1 days versus 4.2 days) in this study, matching the results reported by Arizono et al. [13]. The highly acidic conditions of the human stomach can be challenging for anisakid nematodes to survive because they are known to generally parasitize the nonglandular stomachs of cetaceans, which do not secrete acidic gastric juice [13]. Thus, when they are introduced into human stomachs, they probably try to evade the acidic conditions and penetrate the gastric or intestinal mucosa, causing physical damage. In this study,* A. simplex* (s.s.) started to die earlier than* A. pegreffii*.* A. simplex* (s.s.) started to die at 1 DPI, while* A. pegreffii* started to die at 2 DPI in AGJ. In PBS,* A. simplex* (s.s.) started to die at 3 DPI, while* A. pegreffii* started to die at 6 DPI (Table 3). Some of the individual* A. simplex* (s.s.) L3 used in this study might have been physically damaged or weakened. Nevertheless, the survival rate of* A. simplex* (s.s.) was significantly greater than that of* A. pegreffii* (*P* = 0.001 by the log rank test) (Figure 1). The mean survival time was 6.1 days for* A. simplex* (s.s.) and 4.2 days for* A. pegreffii*. In addition, at 7 DPI, all of the* A. pegreffii* L3 had died in AGJ, while some of the* A. simplex* (s.s.) L3 were still alive in the same conditions (Table 3). Thus,* A. pegreffii* is thought to be more susceptible to acidic condition than* A. simplex* (s.s.).

As discussed above,* A. pegreffii *had sufficiently similar penetration ability to* A. simplex* (s.s.) in the agar block experiment and even higher penetration ability in some conditions, but a shorter mean survival time in acidic conditions. All of these results indicate that* A. simplex* (s.s.) is more invasive and more tolerant of the acidity of human stomachs than* A. pegreffii*, and* A. simplex* (s.s.) can cause problems in human when ingested, as already suggested by many authors [1–6]. Moreover, it is also suggested that* A. pegreffii* can cause problems in human gastrointestinal tracts if they survive, for example, if they were transported into human intestines by peristaltic movement. In Italy, it is reported that gastric anisakidosis represents most of the clinical cases occurring 1–8 h after ingestion, while intestinal anisakidosis represents 4% of the cases developing within a few days [23–25]. In Japan, gastric infection primarily occurs, whereas intestinal disease is more common in Europe [26].

In the animal experiment, the number of* A. simplex* (s.s.) in the stomach was significantly higher than in other body parts at 3 h (Table 4). Our results support that* A. simplex* (s.s.) can cause problems within a few hours after ingestion, as mentioned by many authors [2, 3, 6, 27]. In addition, some of the larvae were found freely in the abdominal cavity or attached to the inner stomach wall, indicating that* A. simplex* (s.s.) has sufficient penetration ability, as already demonstrated by many authors [13, 21, 28]. For* A. pegreffii*, the number in the stomach was significantly higher than in other body parts at 3 h and 6 h. Interestingly, some* A. pegreffii* L3 were found freely in the abdominal cavity or attached to the stomach wall (Table 3) during the experiment, suggesting that* A. pegreffii* also has the ability to penetrate host tissue.

Although we administered 30 individual L3 of* A. simplex* (s.s.) or* A. pegreffii* to each individual rat, not all of the L3 administered were recovered during necropsy. Some of them were thought to be regurgitated or excreted with feces during experimental period. Similar results were obtained by Zuloaga et al. [29], who mentioned that one-third of the administered larvae were not found in their study and suggested that the larvae might have been destroyed and/or evacuated. In addition, there were no significant differences in the number of L3 at each time point between two* Anisakis* species in this study, suggesting that this phenomenon is not species-specific.

In experimental studies using fish and mammalian hosts,* A. pegreffii* L3 had different migration abilities inside the bodies of the hosts, depending on host species [8, 9, 28, 30, 31]. For example, Suzuki et al. [8] demonstrated that* A. pegreffii* showed a lower tendency of penetrating into fish muscles compared to* A. simplex* (s.s.). Furthermore, experimental infections of fish also demonstrated that* A. pegreffii* were recovered within the body cavity and rarely in the body muscle, but* A. simplex* (s.s.) migrated into the body muscle by using rainbow trout (*Oncorhynchus mykiss*) and olive flounder (*Paralichthys olivaceus*) [9]. Probably most of* A. pegreffii* in the fish abdomen are removed with the viscera, which can explain the fewer human cases due to* A. pegreffii*. For mammalian hosts,* A. pegreffii* showed considerable ability to penetrate the gastric mucosa of rats [28], and similar results were also obtained in this study. All of these suggest that* A. pegreffii* can be pathogenic to cause anisakidosis in humans when ingested, like* A. simplex* (s.s.).

The pathological changes in human anisakidosis are thought to occur due to the physical damage to the gastrointestinal tissue during the invasion of the anisakid nematode L3 and the complex interaction between the host immune system and the substances secreted by or contained within the L3 [2]. Although their invasion ability was examined in this study, the roles of chemical substances from these two sibling species during invasion remain to be elucidated. In addition, many factors such as the host specificity of the parasites and the hosts' anatomical and histological structure are thought to be involved in the different pathogenicity of these two* Anisakis* species. These factors are thought to contribute to epidemiological features of anisakidosis worldwide, with their different geographical distribution and fish host range.

## Figures and Tables

**Table 1:** Cumulative penetration rate of *Anisakis  simplex* (s.s.) and *A.  pegreffii* from 2 different agar blocks overlaid with 3 different supernatants. Data are expressed as the average penetration rate ± standard error of five replicates. Means in each column with the same superscript lower-case letters (a–c for *A.  simplex* (s.s.) and d–f for *A.  pegreffii*) are not significantly different (*P* > 0.05). Means in each row with the same subscript upper-case letters (A–C for *A.  simplex* (s.s.) and D–F for *A.  pegreffii*) are not significantly different (*P* > 0.05).

Species	Times after administration	Penetration rate (%) of anisakid nematodes larvae					
		Group A (agar block in PBS)			Group B (agar block in RPMI-1640)		
		PBS	AGJ^1^	RPMI-1640^2^	PBS	AGJ^1^	RPMI-1640^2^
*Anisakis simplex* (s.s.)	1 h	10.0 ± 8.1^a^_A_	10.0 ± 8.1^a^_A_	8.3 ± 3.7^a^_A_	15.0 ± 4.9^a^_A_	5.0 ± 2.0^a^_A_	10.0 ± 6.1^a^_A_
	12 h	25.0 ± 12.9^ab^_A_	38.3 ± 9.0^b^_A_	25.0 ± 12.4^ab^_A_	25.0 ± 7.0^a^_A_	31.7 ± 14.0^ab^_A_	28.3 ± 8.2^ab^_A_
	24 h	30.0 ± 14.1^ab^_A_	41.7 ± 9.1^b^_A_	28.3 ± 11.1^ab^_A_	58.3 ± 12.6^b^_A_	56.7 ± 10.0^bc^_A_	45.0 ± 13.1^bc^_A_
	48 h	51.7 ± 12.2^b^_AB_	43.3 ± 10.0^b^_A_	46.7 ± 10.1^b^_AB_	76.7 ± 7.2^b^_B_	75.0 ± 7.9^c^_B_	71.7 ± 8.6^c^_AB_

*Anisakis pegreffii *	1 h	25.0 ± 5.9^d^_D_	68.3 ± 6.1^d^_F_	40.0 ± 4.9^d^_DE_	50.0 ± 8.3^d^_EF_	33.3 ± 10.9^d^_DE_	53.3 ± 4.3^d^_EF_
	12 h	41.7 ± 2.6^e^_D_	81.7 ± 6.1^d^_E_	46.7 ± 5.7^d^_D_	55.0 ± 7.7^d^_D_	43.3 ± 12.5^d^_D_	66.7 ± 8.3^d^_DE_
	24 h	41.7 ± 2.6^e^_D_	81.7 ± 6.1^d^_F_	48.3 ± 5.5^d^_DE_	63.3 ± 7.7^d^_DEF_	46.7 ± 12.0^d^_D_	71.7 ± 9.7^d^_EF_
	48 h	50.0 ± 3.7^e^_D_	83.3 ± 7.0^d^_E_	51.7 ± 4.1^d^_D_	65.0 ± 6.7^d^_DE_	46.7 ± 12.0^d^_D_	71.7 ± 9.7^d^_DE_

^1^AGJ: artificial gastric juice (pH 1.8, 0.1% pepsin, 0.1% porcine stomach mucin, 0.12% NaCl, 0.02% KCl).

^2^RPMI-1640: RPMI-1640 (pH 4.0, RPMI-1640 with 20% FBS, 1% commercial pepsin).

**Table 2:** Comparison of cumulative penetration of *Anisakis  simplex* (s.s.) and *Anisakis  pegreffii* in 2 different agar blocks overlaid with 3 different supernatants. Data are expressed as the average penetration rate ± standard error of five replicates.

	Supernatant	Times after administration	*A. simplex* (s.s.)	*A. pegreffii *	*P* value^a^
Group A (agar block in PBS)	PBS	1 h	10.0 ± 8.1	25.0 ± 5.9	0.172
		12 h	25.0 ± 12.9	41.7 ± 2.6	0.242
		24 h	30.0 ± 14.1	41.7 ± 2.6	0.439
		48 h	51.7 ± 12.2	50.0 ± 3.7	0.898
	AGJ	1 h	10.0 ± 8.1	68.3 ± 6.1	0.000^*^
		12 h	38.3 ± 9.0	81.7 ± 6.1	0.004^*^
		24 h	41.7 ± 9.1	81.7 ± 6.1	0.007^*^
		48 h	43.3 ± 10.0	83.3 ± 7.0	0.011^*^
	RPMI-1640	1 h	8.3 ± 3.7	40.0 ± 4.9	0.001^*^
		12 h	25.0 ± 12.4	46.7 ± 5.7	0.149
		24 h	28.3 ± 11.1	48.3 ± 5.5	0.144
		48 h	46.7 ± 10.1	51.7 ± 4.1	0.657

Group B (agar block in RPMI-1640)	PBS	1 h	15.0 ± 4.9	50.0 ± 8.3	0.007^*^
		12 h	25.0 ± 7.0	55.0 ± 7.7	0.020^*^
		24 h	58.3 ± 12.6	63.3 ± 7.7	0.744
		48 h	76.7 ± 7.2	65.0 ± 6.7	0.268
	AGJ	1 h	5.0 ± 2.0	33.3 ± 10.9	0.033^*^
		12 h	31.7 ± 14.0	43.3 ± 12.5	0.552
		24 h	56.7 ± 10.0	46.7 ± 12.0	0.538
		48 h	75.0 ± 7.9	46.7 ± 12.0	0.044^*^
	RPMI-1640	1 h	10.0 ± 6.1	53.3 ± 4.3	0.000^*^
		12 h	28.3 ± 8.2	66.7 ± 8.3	0.011^*^
		24 h	45.0 ± 13.1	71.7 ± 9.7	0.140
		48 h	71.7 ± 8.6	71.7 ± 9.7	1.000

^a^by independent *t*-test.

^*^Statistically significant (*P* < 0.05).

**Table 3:** Survival rate (%) of *Anisakis  simplex* (s.s.) and *Anisakis  pegreffii* immersed in 2 different solutions. Data are expressed as the mean survival rate of triplicates ± standard errors.

Species	Days after immersion	PBS^a^	AGJ^b^
*Anisakis simplex* (s.s.)	1	100.0 ± 0.0	83.3 ± 12.0
	2	100.0 ± 0.0	83.3 ± 12.0
	3	83.3 ± 12.0	66.6 ± 6.7
	4	83.3 ± 12.0	56.7 ± 6.7
	5	80.0 ± 15.3	50.0 ± 5.8
	6	80.0 ± 15.3	36.7 ± 3.3
	7	80.0 ± 15.3	3.33 ± 3.3

*Anisakis pegreffii *	1	100.0 ± 0.0	100.0 ± 0.0
	2	100.0 ± 0.0	66.7 ± 3.3
	3	100.0 ± 0.0	33.3 ± 3.3
	4	100.0 ± 0.0	20.0 ± 5.8
	5	100.0 ± 0.0	6.7 ± 6.7
	6	96.7 ± 3.3	3.3 ± 3.3
	7	96.7 ± 3.3	0.0 ± 0.0

^a^PBS: phosphate buffered saline, pH 7.2.

^b^AGJ: artificial gastric juice, pH 1.8.

**Table 4:** The locations of *Anisakis  simplex* (s.s.) and *Anisakis  pegreffii *in rats after necropsy. Means in each column with the same superscript lower-case letters (a–c for *A.  simplex* (s.s.) and d–f for *A.  pegreffii*) are not significantly different (*P* > 0.05). Means in each row with the same subscript upper-case letters (A–C for *A.  simplex* (s.s.) and D–F for *A.  pegreffii*) are not significantly different (*P* > 0.05).

Species	Time after challenge	Number of rats	Number of administered larvae	% of recovered larvae/administered larvae						Number of recovered larvae/administered larvae (%)
				Esophagus	Stomach wall (inner)	Stomach	Stomach wall (outer)	Intestines	Abdominal cavity	
*Anisakis simplex* (s.s.)	3 h	3	30	0.0 ± 0.0^a^_A_	6.7 ± 6.7^a^_A_	60.0 ± 11.6^a^_B_	0.0 ± 0.0^a^_A_	10.0 ± 5.8^a^_A_	10.0 ± 10.0^a^_A_	86.7 ± 13.3^c^
	6 h	3	30	3.3 ± 3.3^a^_A_	3.3 ± 3.3^a^_A_	40.0 ± 15.3^ab^_B_	0.0 ± 0.0^a^_A_	23.3 ± 14.5^a^_AB_	6.7 ± 3.3^a^_A_	76.7 ± 6.7^ab^
	12 h	3	30	0.0 ± 0.0^a^_A_	3.3 ± 3.3^a^_A_	20.0 ± 10.0^bc^_AB_	0.0 ± 0.0^a^_A_	16.7 ± 8.8^a^_AB_	0.0 ± 0.0^a^_A_	40.0 ± 20.8^bc^
	24 h	3	30	0.0 ± 0.0^a^_A_	10.0 ± 5.8^a^_A_	0.0 ± 0.0^c^_A_	0.0 ± 0.0^a^_A_	6.7 ± 3.3^a^_A_	3.3 ± 3.3^a^_A_	20.0 ± 5.8^c^

*Anisakis pegreffii *	3 h	3	30	0.0 ± 0.0^d^_D_	3.3 ± 3.3^d^_D_	50.0 ± 15.3^a^_E_	13.3 ± 8.8^d^_D_	3.3 ± 3.3^d^_D_	3.3 ± 3.3^d^_D_	73.3 ± 8.8^a^
	6 h	3	30	0.0 ± 0.0^d^_D_	6.7 ± 3.3^d^_D_	53.3 ± 14.5^a^_E_	3.3 ± 3.3^d^_D_	10.0 ± 5.8^d^_D_	0.0 ± 0.0^d^_D_	73.3 ± 17.6^a^
	12 h	3	30	0.0 ± 0.0^d^_D_	20.0 ± 10.0^d^_DE_	30.0 ± 11.6^ab^_E_	0.0 ± 0.0^d^_D_	6.7 ± 3.3^d^_D_	10.0 ± 5.8^d^_DE_	66.7 ± 3.3^a^
	24 h	3	30	0.0 ± 0.0^d^_D_	6.7 ± 3.3^d^_DE_	3.3 ± 3.3^b^_D_	6.7 ± 3.3^d^_DE_	0.0 ± 0.0^d^_D_	36.7 ± 23.3^d^_E_	53.3 ± 23.3^a^
